# Leveraging Artificial Neural Networks and Support Vector Machines for Accurate Classification of Breast Tumors in Ultrasound Images

**DOI:** 10.7759/cureus.73067

**Published:** 2024-11-05

**Authors:** Mohammed Othman Abdullah, Yener Altun, Rizgar Maghded Ahmed

**Affiliations:** 1 Department of Statistics, The Institute of Natural and Applied Sciences, Van Yüzüncü Yıl University, Van, TUR; 2 Department of Business Administration, Faculty of Economics and Administrative Sciences, Van Yüzüncü Yıl University, Van, TUR; 3 Department of Statistics and Informatics, College of Administration and Economics, Salahaddin University, Erbil, IRQ

**Keywords:** artificial neural networks (ann), breast cancer classification, machine learning in medical diagnosis, support vector machines (svm), ultrasound imaging

## Abstract

Background and Aim

Breast cancer is a leading cause of cancer-related deaths among women, and ultrasound imaging is crucial for early detection. However, variability in interpretation can affect diagnosis. Therefore, this study compared the performance of artificial neural networks (ANNs) and support vector machines (SVMs) in classifying breast tumors using ultrasound images.

Method

This comparative study was conducted from June 1, 2023, to June 1, 2024, using a convenience sampling method. Data were gathered from the Center for Breast Diseases at Nanakali Hospital in Erbil, Kurdistan Region of Iraq, and a publicly available dataset from Kaggle. ANN and SVM models were then applied to classify the tumors. Statistical analysis was performed using R (R Foundation for Statistical Computing, Vienna, Austria) and IBM SPSS Statistics for Windows, Version 28.0 (Released 2021; IBM Corp., Armonk, New York, United States), with performance metrics such as accuracy, sensitivity, specificity, and Kappa coefficient calculated for both models.

Results

The ANN model achieved an accuracy of 87.78%, with a sensitivity of 86.67% and a specificity of 88.89%. The SVM model demonstrated an accuracy of 86.67%, with a higher specificity of 95.56% but a lower sensitivity of 77.78%. Both models showed substantial agreement between predicted and actual classifications, with Kappa coefficients of 75.56% for ANN and 73.33% for SVM. The mean, skewness, and area were identified as the most important variables for the ANN model, while solidity, circularity, and perimeter were the most critical features of the SVM model.

Conclusions

The results indicated that ANN had a marginally higher accuracy than SVM in classifying breast tumors. It is recommended to further optimize these models for clinical use, improve the integration of machine learning in medical imaging, and expand the dataset to enhance model generalizability and robustness.

## Introduction

Breast cancer remains a leading cause of cancer-related deaths among women worldwide, with an estimated 2.3 million new cases and 685,000 deaths in 2020 [1]. Early detection and accurate diagnosis are crucial for improving patient outcomes and survival rates [2]. In particular, ultrasound imaging has emerged as a valuable tool for breast cancer screening and diagnosis, particularly for women with dense breast tissue or as a complementary modality to mammography [3]. However, the interpretation of ultrasound images is highly dependent on the radiologist's expertise and can be subject to inter-observer variability [4,5]. To address this challenge, recent advancements in artificial intelligence (AI) and machine learning (ML) have shown promise in assisting radiologists with the interpretation of medical images, including ultrasound [6,7]. AI algorithms can analyze large datasets, identify patterns, and provide objective and consistent diagnoses [8]. While existing methodologies such as convolutional neural networks (CNNs) and other machine learning techniques have demonstrated significant progress in breast cancer diagnosis using ultrasound, our approach integrates both artificial neural networks (ANNs) and support vector machines (SVMs) to leverage their respective strengths.

Two prominent ML techniques that have been successfully applied in medical image analysis are ANNs and SVMs [9]. While both methods have distinct advantages, ANNs are inspired by the structure and function of biological neural networks in the human brain [10]. They consist of interconnected nodes (neurons) organized in layers, which can learn from data by adjusting the strength of connections between neurons. ANNs have shown remarkable performance in tasks such as image classification, segmentation, and feature extraction [11]. Specifically in breast ultrasound, ANNs have been employed for tumor detection, classification, and segmentation [12]. SVMs, on the other hand, are based on the principle of finding an optimal hyperplane that separates different classes of data points in a high-dimensional space [13]. SVMs have been widely used in pattern recognition and classification tasks, including medical image analysis [14]. They, too, have demonstrated high accuracy and robustness in classifying breast tumors in ultrasound images [15].

The combination of ANNs and SVMs has the potential to leverage the strengths of both techniques and enhance the accuracy of breast tumor classification in ultrasound images [16]. While ANNs can learn complex patterns and extract meaningful features from the images, SVMs can effectively classify the extracted features into benign or malignant categories [17]. This hybrid approach has shown promising results in previous studies [17,18], highlighting the potential for improving the diagnostic performance of breast ultrasound. Moreover, the integration of AI and ML techniques into clinical practice can assist radiologists in making more accurate and efficient diagnoses, reducing the workload and minimizing human error [19]. By offering a second opinion, these AI-based systems can prioritize suspicious cases and identify subtle patterns that may be overlooked by human observers. This can lead to earlier detection of breast cancer, more personalized treatment plans, and ultimately, better patient outcomes.

However, despite the promising applications of ANNs and SVMs in breast tumor classification, there are still challenges and limitations that need to be addressed. One major challenge is the availability of large, high-quality, and annotated datasets for training and validating the AI models. The performance of ANNs and SVMs heavily relies on the quality and quantity of the training data, and the lack of comprehensive datasets can limit their generalizability and robustness. Additionally, the black-box nature of some AI models, particularly deep learning-based ANNs, can hinder their interpretability and acceptability in clinical practice. For these reasons, physicians may be reluctant to trust the decisions made by AI systems without clear explanations or insights into the reasoning process. Therefore, this study aims to address gaps in breast tumor classification by leveraging ANNs and SVMs for accurate differentiation between benign and malignant tumors using ultrasound images.

## Materials and methods

This was a comparative study conducted from June 1, 2023, to June 1, 2024, using a convenience sampling method. Data were gathered from two sources: the Center for Breast Diseases at Nanakali Hospital in Erbil, Kurdistan Region of Iraq, and a publicly available breast ultrasound image dataset from Kaggle. While these two distinct cohorts differ in terms of geographical origin and clinical settings, the breast ultrasound images from both sources were matched based on key diagnostic features such as tumor size, shape, and tissue density. This study utilized pre-existing anonymized data, both from a public Kaggle dataset and Nanakali Hospital, where patient privacy and confidentiality were ensured. Given that no new data collection involving human participants occurred, formal ethical approval was not required. However, the study was conducted following the ethical principles outlined in the Declaration of Helsinki.

Sample size

The sample size for this study was determined based on the availability of data from the two sources. A total of 300 high-quality ultrasound images were used, each with a resolution of 500 x 500 pixels and a color depth of 24 bits. The decision to use 300 images was due to the limitations of available data and the time-intensive process of manual annotation and verification. Each image was pathologically assessed by specialists and classified as either benign or malignant based on the results of the laboratory analysis. Of these images, 150 were classified as benign and 150 as malignant. Both datasets underwent the same preprocessing procedures to ensure consistency in feature extraction and analysis.

Inclusion and exclusion criteria

Inclusion criteria for the study were breast ultrasound images that were pathologically assessed and diagnosed as either benign or malignant tumors. The images included patients between the ages of 25 and 75 years, regardless of other health conditions. Exclusion criteria involved any images that lacked clear resolution or any images where the tumor classification was ambiguous.

Study tools and data collection

**Figure 1:**
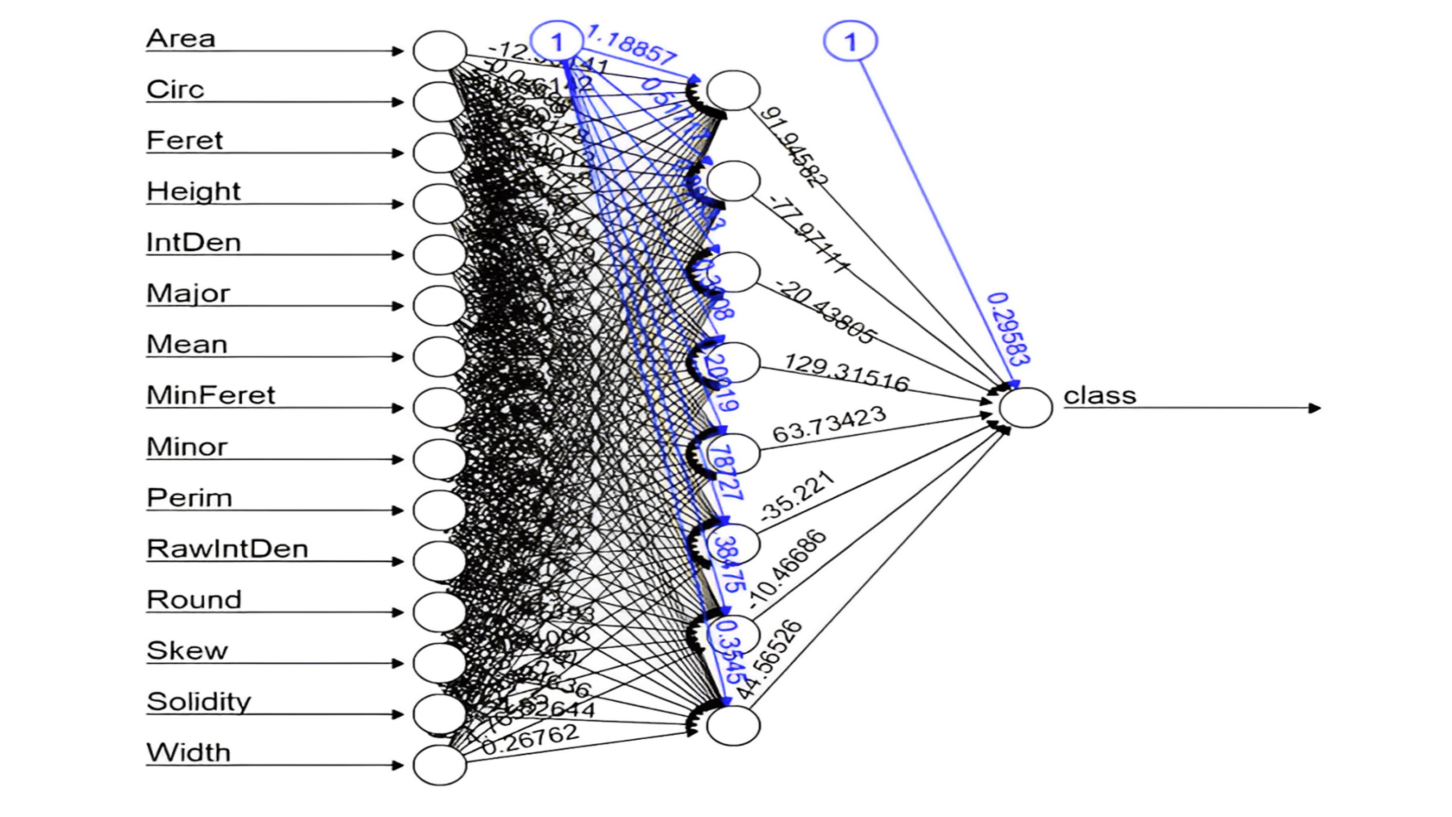
Schematic Diagram of the Artificial Neural Network (ANN) Model for Breast Tumor Classification This diagram illustrates the structure and workflow of the ANN model, highlighting input features, hidden layers, and output classification for differentiating between benign and malignant tumors. RawIntDen: raw integrated density; IntDen: integrated density; Circ: circularity; Feret: Feret diameter; MinFeret: minimum Feret diameter

**Figure 2:**
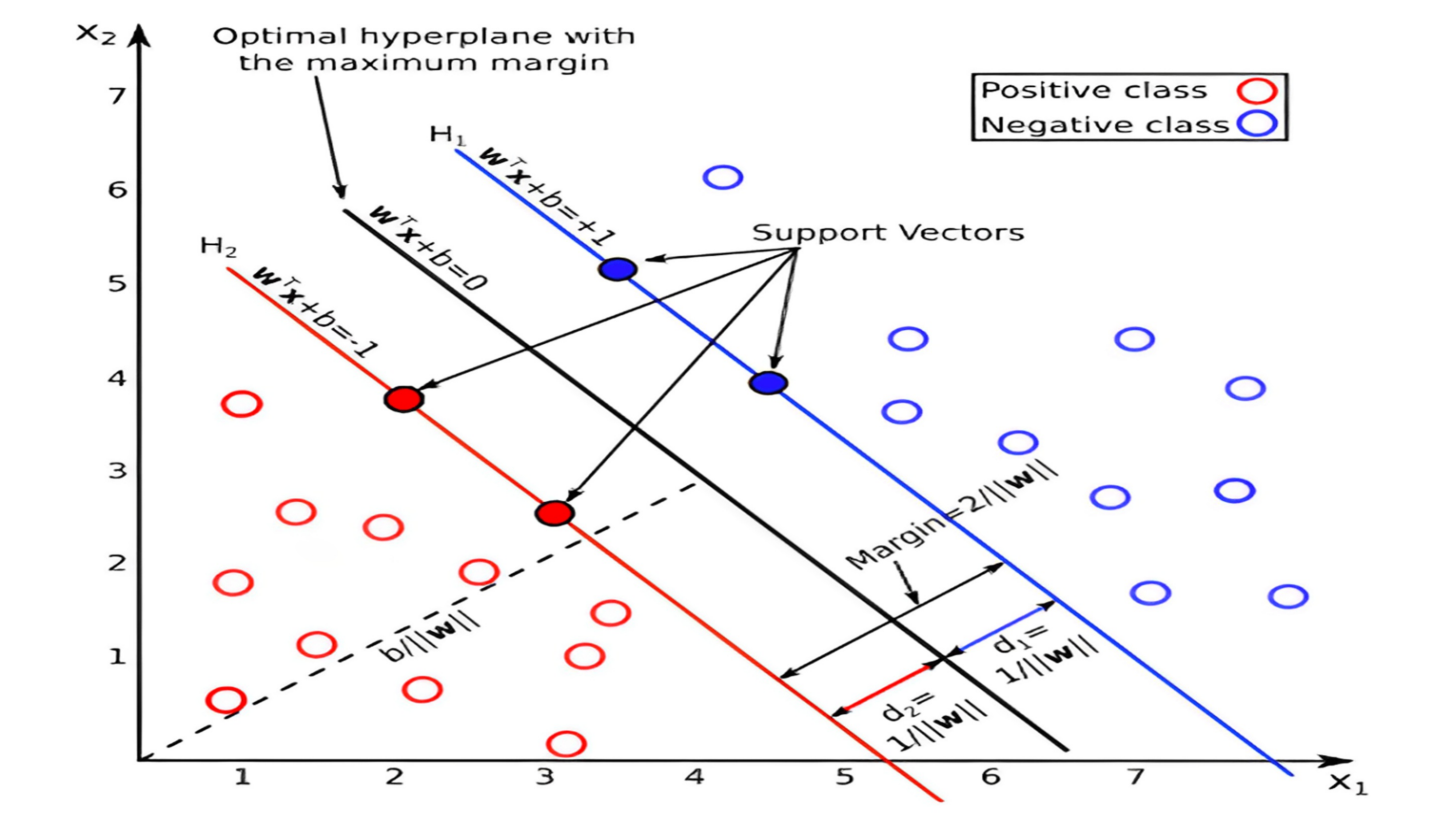
Schematic Diagram of the Support Vector Machine (SVM) Model for Breast Tumor Classification This diagram shows the SVM model's structure, including the input features, support vectors, optimal hyperplane, and classification process used to distinguish between benign and malignant tumors

This study employed two ML models, ANNs and SVMs, to classify breast tumors based on ultrasound image features. Data processing and analysis were conducted using ImageJ software (National Institutes of Health, Bethesda, Maryland, United States) for image segmentation, enabling the extraction of critical tumor characteristics such as area, perimeter, skewness, and solidity. These images were transformed into data points, which were then used as inputs for the classification models. The architecture of the ANN model is illustrated in Figure 1, while Figure 2 shows the SVM model, including its optimal hyperplane and support vectors. Subsequently, statistical and geometric features were extracted from each image, serving as input variables for both models. The dataset was divided into training (70%) and testing (30%) subsets to evaluate model performance. Cross-validation confirmed the reliability and stability of both the ANN and SVM models, as they demonstrated consistent classification accuracy across different data subsets.

Measures

Image Features

The independent variables used in the study were the geometric and statistical features extracted from the ultrasound images. These included the area, which represents the total pixel count of the tumor region, and the perimeter, indicating the length of the tumor boundary. Skewness was used as a measure of asymmetry in the tumor shape, while solidity was calculated as the ratio of the tumor area to its convex hull area. Additional descriptors such as roundness, height, width, and feret diameter provided further details about the tumor’s overall shape and size.

Tumor Classification

The dependent variable was the classification of the tumor as either benign (coded as 0) or malignant (coded as 1).

Statistical Analysis

Data were summarized using means and standard deviations for quantitative variables, and frequencies and percentages for categorical variables. For model evaluation, performance metrics such as accuracy, sensitivity, specificity, and the Kappa coefficient were calculated for both the ANN and SVM models. Additionally, receiver operating characteristic (ROC) curves were used to compare the diagnostic performance of the models. Statistical analysis was conducted using R software version 4.0.5 (R Foundation for Statistical Computing, Vienna, Austria) and IBM SPSS Statistics for Windows, Version 28.0 (Released 2021; IBM Corp., Armonk, New York, United States), focusing on comparing the performance of the ANN and SVM models.

## Results

Confusion matrix for ANN (training data)

Table 1 presents the confusion matrix for the ANN model on the training dataset. The model correctly predicted 104 benign cases and 102 malignant cases, with only four misclassifications (three benign predicted as malignant and one malignant predicted as benign). The model achieved a high accuracy of 98.1%, demonstrating strong performance in identifying both benign and malignant tumors. The sensitivity of 97.14% indicates that the model was very effective in detecting malignant cases, while the specificity of 99.05% shows that it accurately classified benign cases. The Kappa coefficient of 96.19% suggests an excellent level of agreement between the model’s predictions and the actual classifications, reinforcing the model's reliability on the training data.

**Table 1:** Confusion Matrix for Artificial Neural Network (Training Data) Note: The model achieved an accuracy of 98.1%, with a sensitivity of 97.14% and a specificity of 99.05%. The Kappa coefficient, which measures the agreement between predicted and actual classifications, was 96.19%, indicating excellent model performance.

Actual \ Predicted	Benign	Malignant	Total
Benign	104	3	107
Malignant	1	102	103
Total	105	105	210

Confusion matrix for ANN (testing data)

The confusion matrix for the ANN model on the testing data shows that the model correctly predicted 40 benign cases and 39 malignant cases, with 11 misclassifications (six benign predicted as malignant and five malignant predicted as benign). The overall accuracy of the model was 87.78%, demonstrating good performance on unseen data. The sensitivity, at 86.67%, indicates that the model effectively detected malignant cases, while the specificity of 88.89% shows its ability to accurately classify benign cases. The Kappa coefficient of 75.56% suggests a substantial agreement between the model’s predictions and the actual classifications, indicating reliable performance in the testing phase. For a more detailed analysis, refer to Table 2.

**Table 2:** Confusion Matrix for Artificial Neural Network (Testing Data) Note: The model achieved an accuracy of 87.78%, with a sensitivity of 86.67% and a specificity of 88.89%. The Kappa coefficient was 75.56%, indicating substantial agreement between predicted and actual classifications.

Actual \ Predicted	Benign	Malignant	Total
Benign	40	6	46
Malignant	5	39	44
Total	45	45	90

Confusion matrix for SVM (training data)

The confusion matrix for the SVM model on the training data indicates that the model correctly classified 98 benign cases and 90 malignant cases, with 22 misclassifications (15 benign predicted as malignant and seven malignant predicted as benign). The model achieved an accuracy of 89.52%, reflecting good classification performance on the training data. With a sensitivity of 85.71%, the model was reasonably effective in detecting malignant cases, while a specificity of 93.33% shows that it performed well in correctly identifying benign cases. The Kappa coefficient of 79.05% suggests substantial agreement between the predicted and actual classifications, indicating a reliable model performance during training. For a more detailed analysis, refer to Table 3.

**Table 3:** Confusion Matrix for Support Vector Machine (Training Data) Note: The model achieved an accuracy of 89.52%, with a sensitivity of 85.71% and a specificity of 93.33%. The Kappa coefficient was 79.05%, indicating substantial agreement between predicted and actual classifications.

Actual \ Predicted	Benign	Malignant	Total
Benign	98	15	113
Malignant	7	90	97
Total	105	105	210

Confusion matrix for SVM (testing data)

The confusion matrix for the SVM model on the testing data shows that the model correctly classified 43 benign cases and 35 malignant cases, with 12 misclassifications (10 benign predicted as malignant and two malignant predicted as benign). The model achieved an overall accuracy of 86.67%, indicating good performance in classifying unseen data. With a sensitivity of 77.78%, the model was moderately effective at identifying malignant cases, while its high specificity of 95.56% highlights its strong ability to correctly classify benign cases. The Kappa coefficient of 73.33% indicates substantial agreement between the predicted and actual classifications, reflecting reliable performance on the testing data (Table 4).

**Table 4:** Confusion Matrix for Support Vector Machine (Testing Data) Note: The model achieved an accuracy of 86.67%, with a sensitivity of 77.78% and a specificity of 95.56%. The Kappa coefficient was 73.33%, reflecting substantial agreement between the predicted and actual classifications.

Actual \ Predicted	Benign	Malignant	Total
Benign	43	10	53
Malignant	2	35	37
Total	45	45	90

Performance comparison between ANN and SVM (testing data)

The performance comparison between ANN and SVM on the testing data shows that both models performed well, with ANN achieving a slightly higher accuracy (87.78%) than SVM (86.67%). ANN also demonstrated higher sensitivity (86.67%) in detecting malignant cases, whereas SVM had superior specificity (95.56%) in correctly classifying benign cases. The Kappa coefficient for both models indicates substantial agreement between predicted and actual classifications, with ANN at 75.56% and SVM at 73.33%. The error rates were similar, with ANN showing a marginally lower error rate (12.22%) compared to SVM (13.33%), indicating that both models are robust in their classification tasks. Table 5 gives more detailed metrics.

**Table 5:** Performance Comparison Between ANN and SVM (Testing Data) ANN: artificial neural network; SVM: support vector machines

Metric	ANN	SVM
Accuracy	87.78%	86.67%
Sensitivity	86.67%	77.78%
Specificity	88.89%	95.56%
Kappa Coefficient	75.56%	73.33%
Error Rate	12.22%	13.33%

Case examples

**Figure 3:**
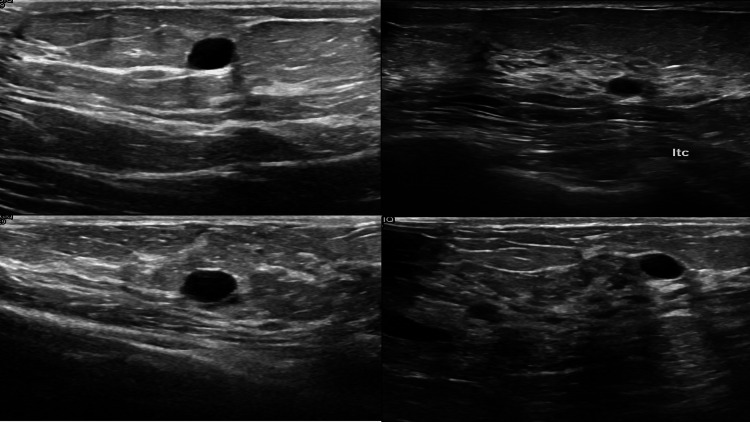
Representative Ultrasound Image of a Benign Tumor Case This presents an example of a benign tumor from the dataset, highlighting key extracted features such as area, perimeter, skewness, and solidity, which were used for classification in the ANN and SVM models. ANN: artificial neural network; SVM: support vector machines

**Figure 4:**
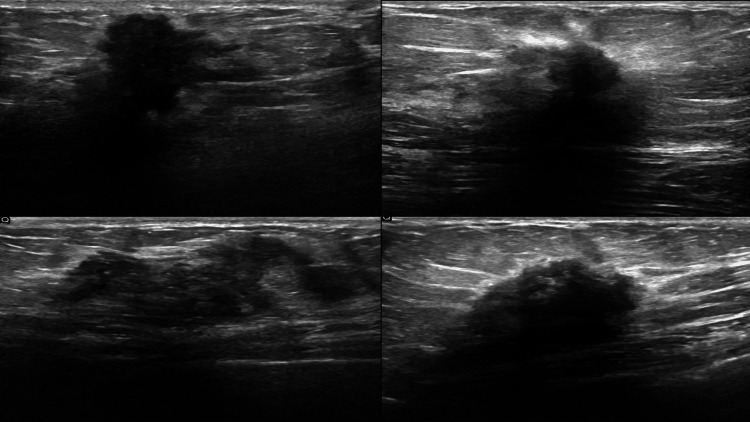
Representative Ultrasound Image of a Malignant Tumor Case This displays an example of a malignant tumor from the dataset, showing essential extracted features such as area, perimeter, skewness, and solidity, utilized for classification by the ANN and SVM models. ANN: artificial neural network; SVM: support vector machines

To illustrate the data used in this study, we provide representative examples of both benign and malignant tumors. Figure 3 shows a sample benign tumor case, highlighting key extracted features such as area, perimeter, skewness, and solidity, which contributed to the classification. Figure 4 presents a malignant tumor example, emphasizing the attributes that helped differentiate it from benign cases.

Variable importance for ANN model

The variable importance analysis for the ANN model reveals that the mean is the most influential feature, with a 100% importance score, followed by skewness (88.42%) and area (80.00%). Other important variables include raw integrated density (RawIntDen) at 78.22% and height at 76.56%. Features such as integrated density (IntDen) and circularity (Circ) also play significant roles, with importance values of 75.00% and 66.32%, respectively. Lower-ranked variables like Feret diameter (48.62%) and solidity (32.23%) still contribute, albeit to a lesser degree. Further insights into the ranking of each variable can be found in the Table 6.

**Table 6:** Variable Importance for ANN Model RawIntDen: raw integrated density; IntDen: integrated density; Circ: circularity; Feret: Feret diameter; MinFeret: minimum Feret diameter; ANN: artificial neural network

Variable	Importance (%)
Mean	100.00
Skew	88.42
Area	80.00
RawIntDen	78.22
Height	76.56
IntDen	75.00
Circ	66.32
Minor	62.32
Feret	48.62
Major	47.21
Round	46.23
Perimeter	46.01
Solidity	32.23
Width	16.44
MinFeret	12.33

Variable importance for SVM model

The variable importance analysis for the SVM model indicates that solidity is the most critical feature, with an importance score of 100%, followed by circularity (Circ) at 94.77% and perimeter at 80.06%. Other notable variables include height (74.48%) and RawIntDen and IntDen, both with an importance score of 71.94%. Variables like Feret siameter (69.42%) and area (63.17%) also play a significant role. Lower-ranked features, such as skewness (18.61%) and mean (15.73%), contribute less to the classification performance. A full breakdown of variable rankings is given in Table 7.

**Table 7:** Variable Importance for SVM Model RawIntDen: raw integrated density; IntDen: integrated density; Circ: circularity; Feret: Feret diameter; MinFeret: minimum Feret diameter; SVM: support vector machine

Variable	Importance (%)
Solidity	100.00
Circ	94.77
Perimeter	80.06
Height	74.48
RawIntDen	71.94
IntDen	71.94
MinFeret	69.83
Feret	69.42
Width	66.08
Area	63.17
Major	61.66
Minor	61.25
Skew	18.61
Mean	15.73
Round	0.00

## Discussion

The present study aimed to compare the performance of ANNs and SVMs in classifying breast tumors using ultrasound images. The results indicated that ANN had a marginally higher accuracy than SVM in classifying breast tumors. This comparison is particularly important given the critical role of early breast cancer detection, a leading cause of cancer-related deaths among women worldwide. Early detection significantly improves patient outcomes. Ultrasound imaging, being non-invasive and widely utilized for breast tumor diagnosis, offers promise but comes with challenges in accurate interpretation. In this context, MK algorithms like ANNs and SVMs have shown potential in aiding radiologists with tumor classification [20]. However, limited research directly compares their performance using ultrasound images, creating a gap that this study aimed to fill. Therefore, evaluating these algorithms in terms of classification performance was a key objective of the study.

The ANN model demonstrated strong performance in classifying both benign and malignant breast tumors on the training data, with high accuracy and few misclassifications. These results align with previous studies highlighting the effectiveness of ANNs in breast tumor classification across various imaging modalities [21,22]. Moreover, the ANN's ability to generalize to unseen data during testing underscores its clinical potential, although the increased misclassifications during this phase suggest that further refinement is needed to optimize the model. The SVM model also exhibited strong performance, particularly in identifying benign tumors. This is consistent with prior findings showing SVMs to be effective in distinguishing between benign and malignant breast lesions [17,23]. However, while the SVM model showed high specificity for benign tumors, its lower sensitivity in detecting malignant cases compared to the ANN model highlights a need for further optimization.

The comparison of the two models revealed that ANN had a slight advantage in terms of overall accuracy and sensitivity, particularly in detecting malignant cases. This result mirrors a previous study where ANNs outperformed SVMs in various medical classification tasks [24]. Nevertheless, the SVM’s superior performance in classifying benign tumors suggests that it may reduce unnecessary biopsies, thus minimizing patient anxiety. Additionally, both models demonstrated substantial agreement with actual classifications, suggesting their potential as valuable clinical decision-support tools. Furthermore, analysis of feature importance for both models highlighted the key variables influencing classification results. For the ANN model, features such as mean, skewness, and area were most significant, while for the SVM model, solidity and circularity played critical roles. These findings are in line with earlier research identifying texture and morphological features as important predictors of breast tumor malignancy [25].

The insights gained from the feature importance analysis offer valuable guidance for future research and model refinement. By understanding which features most significantly contribute to classification accuracy, targeted imaging protocols can be developed to improve diagnostic performance. For instance, the ANN model's reliance on features like mean, skewness, and area suggests that enhancing image resolution and capturing these specific characteristics could further bolster its accuracy. Similarly, for the SVM model, focusing on features such as solidity and circularity could help improve its sensitivity, particularly in detecting malignant cases. Therefore, refining imaging techniques to highlight these critical features could enhance both models' diagnostic capabilities. This focus on feature-driven improvements not only holds promise for enhancing the models' performance but also provides a pathway for developing more personalized and precise diagnostic tools in clinical settings.

Despite these promising results, the study has certain limitations. For example, the relatively small sample size and use of two datasets only may affect the generalizability of the findings. Future research should aim to validate these results using larger, multi-institutional datasets to better assess the robustness of the models across different populations and imaging protocols. Moreover, the retrospective nature of the study and lack of external validation could introduce biases, underscoring the need for prospective studies with independent validation cohorts to evaluate the models' clinical utility in real-world settings.

## Conclusions

The results showed that the ANN exhibited marginally higher accuracy than the SVM in classifying breast tumors, suggesting its potential for slightly improved performance in medical image interpretation. To optimize these models for clinical use, continuous refinement of both ANN and SVM algorithms is essential to enhance their diagnostic accuracy. Additionally, improving the integration of ML into medical imaging workflows can assist radiologists in decision-making, thereby reducing human error and variability. Expanding the dataset to encompass a wider range of cases will further enhance model generalizability and robustness, making these AI tools more reliable across diverse populations and clinical settings. Future research should also explore hybrid models that combine the strengths of different ML techniques to maximize diagnostic efficiency and accuracy.
